# What next after GDP-based cost-effectiveness thresholds?

**DOI:** 10.12688/gatesopenres.13201.1

**Published:** 2020-11-30

**Authors:** Y-Ling Chi, Mark Blecher, Kalipso Chalkidou, Anthony Culyer, Karl Claxton, Ijeoma Edoka, Amanda Glassman, Noemi Kreif, Iain Jones, Andrew J. Mirelman, Mardiati Nadjib, Alec Morton, Ole Frithjof Norheim, Jessica Ochalek, Shankar Prinja, Francis Ruiz, Yot Teerawattananon, Anna Vassall, Alexander Winch

**Affiliations:** 1 Center for Global Development, London, SW1P 3SE, UK; 2 National Treasury, Pretoria, 0002, South Africa; 3 Department of Infectious Disease Epidemiology, Imperial College London, London, SW7 2AZ, UK; 4 Centre for Health Economics, Department of Economics and Related Studies, University of York, York, YO10 5DD, UK; 5 School of Public Health, Wits University, Parktown, 2193, South Africa; 6 Center for Global Development, Washington, D.C., 20036, USA; 7 Sightsavers, Haywards Health, RH16 3BW, UK; 8 Faculty of Public Health, Department of Health Policy and Administration, Universitas Indonesia, Depok, Indonesia; 9 University of Glasgow, Glasgow, UK; 10 BCEPS, Department of Global Public Health and Primary Care, University of Bergen, Bergen, Norway; 11 Department of Community Medicine & School of Public Health, Post Graduate Institute of Medical Education and Research, Chandigarh, 160 012, India; 12 Health Intervention and Technology Assessment Program, Ministry of Public Health, Thailand, Nonthaburi, 11000, Thailand; 13 Department of Global Health and Development, Faculty of Public Health and Policy, London School of Hygiene and Tropical Medicine, London, WC1H 9SH, UK

**Keywords:** Cost-effectiveness thresholds, cost-effectiveness analysis, health opportunity cost, priority setting

## Abstract

Public payers around the world are increasingly using cost-effectiveness thresholds (CETs) to assess the value-for-money of an intervention and make coverage decisions. However, there is still much confusion about the meaning and uses of the CET, how it should be calculated, and what constitutes an adequate evidence base for its formulation. One widely referenced and used threshold in the last decade has been the 1-3 GDP per capita, which is often attributed to the Commission on Macroeconomics and  WHO guidelines on Choosing Interventions that are Cost Effective (WHO-CHOICE). For many reasons, however, this threshold has been widely criticised; which has led experts across the world, including the WHO, to discourage its use. This has left a vacuum for policy-makers and technical staff at a time when countries are wanting to move towards Universal Health Coverage **. **

This article seeks to address this gap by offering five practical options for decision-makers in low- and middle-income countries that can be used instead of the 1-3 GDP rule, to combine existing evidence with fair decision-rules or develop locally relevant CETs. It builds on existing literature as well as an engagement with a group of experts and decision-makers working in low, middle and high income countries.

## Introduction

Public payers around the world are increasingly using Health Technology Assessment (HTA) to inform resource allocation decisions ( Leech *et al.*, 2018; MacQuilkan *et al.*, 2018; Tantivess *et al.*, 2017; World Health Organization, 2015). These decisions are often based on evidence of the expected additional intervention costs and health benefits summarized as the incremental cost-effectiveness ratio (ICER). This is a measure of the value of resources that are actually needed in a specific location and at a specific time to produce one unit of health (most commonly a Quality Adjusted Life Year – QALY- or a Disability Adjusted Life Year – DALY averted). ICERs can be used to compare competing interventions or can be evaluated against a pre-defined decision rule referred to as a Cost-Effectiveness Threshold (CET). The CET sets, on average, the *maximum* financial investment a public payer will commit to generate a unit of health ( Cameron *et al.*, 2018) and is typically used alongside other information to inform decisions around resource allocation in health, particularly around the introduction of new treatments and benefits.

The use of CETs (and ICERs) is typically associated with a goal of health maximisation. They can also, however, be used in conjunction with other criteria. For instance, in Norway a CET was used to maximise both health together with a fair distribution of health ( Ottersen *et al.*, 2016). While health system objectives might vary between jurisdictions, maximising health is one aspiration that we assume is widely shared within and across jurisdictions ( Culyer, 2016).

There is still much confusion about the meaning and uses of the CET, how it should be calculated, and what constitutes adequate evidence base for its formulation ( Ochalek *et al.*, 2015). There are three broad bases on which CETs that are used by public payers are set: willingness to pay (WTP), precedence and opportunity cost (for further discussion, see Santos *et al.,* 2018 and Vallejo-Torres *et al.,* 2016). However, a commonly used approach following none of the three bases is to set the CET at 1-3 times GDP per capita, including in Low and Middle-Income Countries (LMICs). It is often attributed to the Commission on Macroeconomics, and was later adopted in the WHO guidelines on Choosing Interventions that are Cost Effective (WHO-CHOICE) ( World Health Organization Commission on Macroeconomics and Health, 2001). Using this approach, an intervention that averts one DALY for a cost less than GDP per capita is considered “very” cost effective, but an intervention could still count as cost-effective if the ICER did not exceed three times GDP per capita. One rationale behind the rule is that GDP per capita is a proxy for earnings ( Robinson *et al.*, 2017). In other words, if an intervention averts a DALY at less than one GDP per capita, then its return on investments in the wider economy (through increased labour productivity) would offset its implementation costs. Because of its simplicity of use and interpretation, the 1-3 GDP threshold has gained much popularity in recent years. A recent review of cost-effectiveness analyses (CEA) found that 66% of published research studies between 2000 and 2015 in LMICs used a GDP-based CET ( Leech *et al.*, 2018) ^i^.

The 1-3 GDP per capita criterion in the Commission on Macroeconomics and Health report was, however, never intended to be used to determine CETs. It was to be used to value health in benefit-cost analyses (e.g. to make the case for resource allocation to health as opposed to other sectors) ( Robinson *et al.*, 2017). Moreover, GDP-based CETs have no direct relation to a country’s healthcare budget, technical capacity, population preferences or social values ( Marseille *et al.*, 2015). Several experts have also shown that GDP-based CETs can lead to the adoption of interventions that are not in practice locally affordable ( Marseille *et al.*, 2015). One may argue that there is no harm in *casting the net wide* by setting a high CET. This is false, however, because spending health resources inevitably creates opportunity costs. Health opportunity costs arise because resources committed to one intervention are no longer available to fund alternative, perhaps more cost-effective, interventions. As a result, allocating resources to an intervention that would not be included by reference to a more realistic CET can paradoxically result in a loss of health and increase in avoidable deaths, by displacing more health than it creates ( Revill *et al.*, 2018). Health opportunity costs are higher in LMICs because spending on the *wrong* interventions might deplete a country’s resources to pay for affordable and effective interventions. In stylized terms, in a country where a life could be saved by spending $1000 dollars, the misallocation of that same $1000 will cause a death.

These considerations have contributed to a growing unease with the use of GDP-based CETs, especially in LMICs, given how widely they are used in research ( Leech *et al.,* 2018). More recently, several experts, including some at the WHO where the practice has been referenced for decades, have advised against the use of GDP-based CETs as a sole decision rule ( Bertram *et al.*, 2016).

The retraction of the 1-3 GDP rule leaves a vacuum for policy-makers and technical staff at a time when LMICs are aspiring to Universal Health Coverage (UHC) and making decisions that will set the path of health policy and spending for decades to come. As others have observed ( Global Burden of Disease Health Financing Collaborator Network, 2019), mobilising additional resources for health is a lengthy and challenging process; making decision rules such as CETs to ensure adequate spending all the more important. An appropriately set CET could support the realisation of the most generous possible form of UHC by ensuring that existing resources are primarily directed toward cost-effective interventions.

In addition, countries have been encouraged to develop in-country capacity and institutions to support the use of HTA in decision-making. This is fully supported by the WHO and is reflected in the World Health Assembly WHA67/23 resolution ( World Health Organisation, 2014). In several countries (e.g. Indonesia, Kenya, Ghana, South Africa, India and the Philippines), plans to institutionalise HTA or set up HTA committees were announced in official communications ( Addo, 2019; Authority of the Republic of Kenya, 2018; Congress of the Philippines, 2019; Ministry of Health and Family Welfare, 2019; Sharma *et al*., 2020). These committees and agencies are likely to include CETs as part of their decision-making processes.

The theory of CETs is discussed elsewhere and reviews document how countries with existing CETs have defined them ( Cameron *et al.*, 2018; Santos *et al.*, 2018; Thokala *et al.*, 2018; Vallejo-Torres *et al.*, 2016). Shortcomings of existing approaches to CETs, not exclusively focusing on GDP-based CETs, have also been discussed ( Bertram *et al.*, 2016; Marseille *et al.*, 2015; Newall *et al.*, 2014; Vallejo-Torres *et al.*, 2016). What is missing is the creation of practical alternatives to the use of GDP-based thresholds. This article seeks to address this gap by offering five practical options for developing locally relevant CETs (i.e. ones that are informed by local data) or utilising evidence (existing, complementary to CEA) to support decision-makers facing urgent resource allocation decisions.

The paper builds on a consultation between academics, country technical staff and global donors from high, middle, and low income countries (although not representative), organised by the International Decision Support Initiative (Secretariat based in the UK), Centre for Health Economics (University of York) and the Health Intervention and Technology Assessment Program (Thailand), at the Rockefeller Foundation Bellagio Centre between December 3-7, 2018. The group spent three and a half days discussing the role of CETs in achieving UHC in LMICs ^ii^. Our conversation focussed on supply-side approaches. There is one major reason for this. The willingness-to-pay approach raises question of whose willingness matters, a political matter on which we do not feel able to advise, and can also lead to the use of aspirational CETs that are not connected to budget constraints or health opportunity costs. The supply-side approach by contrast is focussed on the resources available, how they are currently used, and what is most likely to be sacrificed when they are used in one way rather than another. These are concrete matters that decision-makers face on a daily basis.

During this meeting, our group collectively defined five options that can replace the GDP based threshold (in the absence of a formal CET) when decision-makers are faced with a new intervention that they need to consider. We have ordered the options from the least to the most resource and data intensive, with option 5 being a within-country empirical estimation of supply-side CET that reflects health opportunity costs. The options are not mutually exclusive: it is possible to combine several approaches according to context and need.

## Option 1. Use existing estimates of national health opportunity cost thresholds derived from cross-country data

Two recent contributions ( Ochalek *et al.*, 2018; Woods *et al.*, 2016) provide estimates of health opportunity costs (i.e. what is given up as a consequence of introducing an intervention), country by country, for a number of LMICs. Woods *et al.* (2016) extrapolate health opportunity costs in LMICs from UK estimates produced by Claxton *et al.* (2015) by applying data on the income elasticity of the value of health to generate ranges of cost per QALY gained estimates in LMICs. Ochalek *et al.* (2018) expand country estimates from Bokhari *et al.* (2007) on the effect of changes in health expenditure on health outcomes and, following Claxton *et al.* (2015), apply country-specific data on health expenditure, epidemiology and demography to calculate a range of cost per DALY averted thresholds estimates in LMICs. Country by country estimates are available in their supplemental materials. To date, those two studies are the best attempts to estimate health opportunity costs using cross-country data sources, despite methodological caveats ^iii^.

Although adopting different approaches, both Woods *et al.* (2016) and Ochalek *et al.* (2018) estimate CETs averaging ^iv^ roughly half of GDP per capita, albeit with a substantial range. On this basis, if a GDP based rule is applied, it would probably be a secure rule to deem as cost-ineffective all interventions averting 1 DALY at more than 1 GDP per capita. Half of GDP per capita is more in accord with countries’ realities than the 1-3 GDP per capita, and could be used as an interim rule of thumb rather than the 1-3 GDP rule. A handful of recent papers have already started using half of GDP per capita ^v^. Francke *et al.* (2016) estimated the cost-effectiveness of diagnostic of HIV infection in early infancy in South Africa, and based on ‘ *emerging literature’*, used a CET of half of GDP per capita. Other studies also used such a CET, although none provided a justification for doing so ( Bilcke *et al.*, 2019; Campos *et al.*, 2018; Mezei *et al.*, 2018). Finally, half of GDP per capita was also referenced in the Disease Control Priorities 3 (DCP3) as an example CET in highly resource constrained countries ( Watkins *et al.*, 2017). Half of GDP per capita will lead to underestimating or overestimating health opportunity costs in roughly half of LMICs ( Ochalek *et al*., 2020b).

## Option 2. Use existing evidence from other settings

Short cuts can sometimes offer an informed way forward when decision-makers are uncertain about appropriate methodologies or lack the necessary skill and evidence base for more sophisticated procedures. One shortcut could be to look at evidence elsewhere by asking the following questions:

- **Have regulatory authorities such as the Food and Drug Administration licensed this product and for which indication?** This question can first identify ‘wasted buys’ ( *i.e.* interventions that have a harmful or non-beneficial effect for a patient) if the intervention under consideration was not licensed (for the right indication), without identifying a CET or conducting a CEA. For instance, a review of the Romanian procurement decisions found that bevacizumab was being used for the treatment of metastatic breast cancer, despite having been withdrawn from FDA approval for this indication ( Lopert *et al.*, 2013; US Food & Drug Administration, 2011). Other agencies in charge of ensuring the safety, efficacy and security of drugs and products can be considered.
- **Was this rejected for funding elsewhere**? The National Institute for Health and Care Excellence (NICE) in the UK publishes its technology appraisal guidance, which contains information on the technology under consideration and the accompanying recommendations. NICE’s **negative** recommendations can be a useful starting point since interventions not recommended in a high-income country are very unlikely to be appropriate choices in LMICs unless it was thought that the United Kingdom’s health system and financial capacity is very different from the country under consideration. On the other hand, a NICE positive recommendation is not to be followed slavishly in LMICs, because of differential health opportunity costs.

CEA estimates are not always transferrable across settings, and there is little guidance on how to make decisions about suitability of estimates in a local context ( Drummond *et al.,* 2009). Nonetheless, if an intervention was found not to be cost-effective in a high-income setting, then it is unlikely to be cost-effective in an LMIC; and it would need important differences in disease epidemiology, intervention costs or health state preferences to warrant adoption in an LMICs.

## Option 3. ICERs and budget impact to inform cost-effectiveness and affordability

Budget impact analyses (BIA) can also support decision-makers in determining whether an intervention is affordable to the country ( Bilinski *et al.*, 2017). BIA is a method of assessing predicted short-term changes in expenditure were a new intervention were introduced. It reflects not only the total cost of its introduction, but also the coverage and uptake rates, as well as potential new health costs (or savings) ( Sullivan *et al.*, 2014). There is often a disconnect between the cost-effectiveness of an intervention, and its affordability to a country ( Bilinski *et al.,* 2017; Howdon *et al.,* 2019; Lomas, 2019; Wiseman *et al.,* 2016). Presenting BIA alongside CEA can ensure that decision-makers can anticipate the resource implications of a new intervention for the allocation of their budget ( Mohara *et al.*, 2012). For instance, treatment of Hepatitis C was found to be cost-effective in many settings, but providing universal access to all eligible patients would have significant resource implications, even in middle-income countries ( Urrutia *et al.*, 2016). In the United Kingdom, treatment of Hepatitis C was found to be cost-effective but this decision was found to be controversial due its budget impact ( Lomas *et al.*, 2018).

Even in countries where a CET is used to inform policy, there is growing consideration of BIA. In Thailand, the ICER is presented alongside budget impact. For instance, the inclusion of Imiglucerase for Gaucher disease type 1 was approved due to the low budget impact, equity concerns and disease severity (terminal condition) even though the treatment was well above the Thai CET ( Leelahavarong, 2019). Since 2017, budget impact for the first three years of use is assessed in the United-Kingdom. If it exceeds a certain threshold for the entire National Health Service (currently £20 million), a phased implementation or price negotiation with manufacturers is initiated ( National Institute for Health and Care Excellence, 2018). However, Bilinski *et al.* (2017) report that fewer than 3% of the 384 published CEA included in their review contained a full report of BIA.

## Option 4. A league table for Health Benefits Package design

A league table is a list of health interventions in order of their ICER. It can be a useful approach to allow decision-makers to appraise a wide range of interventions in one summary table.

When considering a new inclusion to an existing Health Benefits Package (HBP), a league table can be used to identify the least cost-effective intervention that has been funded under the HBP, which can serve as a benchmark to infer what the maximum investment the country was willing to commit to producing an additional unit of health when developing the HBP – or to initiate more detailed evaluation of its likely cost-effectiveness. In other words, the league table helps identify a proxy of the *shadow CET*. This approach can be appropriate if an existing package of services is available in the country and ICERs can be derived for a reasonable number of the interventions included in this package. Using this method, decision-makers could gain confidence that a new entrant would not be included unless it produced more health benefit than the least cost-effective intervention already covered.

On the other hand, when developing an HBP *de novo*, a league table can also be used to set a CET when combined with data on coverage and utilisation. For this, the budget envelope will need to be defined from the onset. This may not be easy, especially in countries where contributions from external partners is significant. For instance, in a study in Malawi from Ochalek *et al.* (2018), the authors highlight that donor funds (often off budget, disbursed through conditionalities) make up 70% of total health expenditure. This creates uncertainties on how the budget line is set (the authors calculate the budget line considering all funding, regardless of the source).

In this option, the budget line determines the CET: a league table is constructed in descending order of cost-effectiveness, and estimates of utilisation are used to calculate the budget impact for each intervention. Culyer (2016) uses a metaphor of a *bookshelf* of healthcare interventions, in which each book is ranked according to its height ( *i.e.,* its effectiveness-cost ratio) and the thickness of the book represents the cost of providing the intervention ( *i.e.*, the budget impact). The threshold corresponds to the least cost-effective intervention affordable to the country before the (fixed) budget is exhausted. This approach was reported to be implemented in Oregon’s Medicaid scheme in the 1990s to define health benefits (although a review ( Tengs *et al.*, 1996) later showed that there was no correlation between the final benefits list and the economic literature or Oregon’s own cost-effectiveness data).

While the data requirements for this option appear be high to some, they can be much reduced by using expert opinion and international evidence to identify a narrower range of likely candidates for more detailed evaluation. This enables decision makers to focus their attention on a manageable number of possible interventions together with their relevant uncertainties. One challenge in LMICs is the low availability of evidence on cost-effectiveness, as well as differences in the methodological specifications employed in studies ( Drummond *et al.*, 1993; Mauskopf *et al.*, 2003). Comparing them usefully requires local epidemiological and economic skills. However, there are several global sources which can be compiled to inform country level estimates: DCP3, the TUFTS GHCEA Registry and WHO-CHOICE. These sources were used by the Ochalek *et al.* (2018) study, although important data gaps remained and were highlighted as a limitation to the study. In Ethiopia, a similar approach was used to develop the Essential Health Services Package ( Ministry of Health Ethiopia, 2019). It is worth noting that WHO-CHOICE present average cost-effectiveness ratios (ACERs) instead of incremental ones ( Arnold *et al.*, 2019). This is sometimes raised as a concern because average cost-effectiveness ratios compare interventions to a *doing nothing* scenario, which is only very rarely an appropriate comparator ( O’Day & Campbell, 2016).

## Option 5. Estimating a health opportunity cost CET using within-country data

Notwithstanding the different approaches to defining a CET, this group found that defining CETs based on health opportunity costs using within-country data was particularly suitable in LMICs given the high opportunity costs created by severe budget constraints. However, each country may want to develop its own national CET, relevant to their own situation.

Relying on health opportunity cost estimates makes it possible to estimate whether the health gains produced by an intervention are greater than the health lost from displacing other interventions in other parts of the health system ( Claxton *et al.*, 2015). This approach derives from the goal of health maximisation. Where health must be sacrificed to improve distributional outcomes (or to meet goals other than health maximisation), a health opportunity cost CET can help quantifying the trade-off. Unlike the WTP method, which bears no link to public budgets, the health opportunity cost method calibrates the CET against the reality of local budget constraints ( Brouwer *et al.*, 2019; Leech *et al.*, 2018).

The seminal works of Claxton *et al.* (2015) in the UK pioneered the estimation of health opportunity costs CETs. They used a very detailed programme budgeting data from the English National Health Service (NHS) to estimate a health opportunity cost CET, which was much lower (£12,936) than the one then applied (£20,000-30,000 and up to £100,000 in certain cases).

Attempts to apply a similar estimation framework have been made in China, Indonesia, India and the Republic of South Africa ( Edoka & Stacey, 2020; Ochalek *et al*., 2020), although with different methods and data (given the paucity of the latter in LMICs). These estimates have two parts: estimating the elasticities of health outcomes with regard to health expenditure using an econometric analysis, and translating the elasticity estimates to health opportunity cost thresholds. In order to estimate health spending elasticities, data on health expenditure and health outcomes ( *e.g.* mortality rates, DALYs or QALYs – and ideally age and gender specific) at a low level of aggregation (e.g. local health authorities or districts and provinces) is required. Ideally, these data will need to be collected across several time periods/years. Furthermore, the estimation of health spending elasticities could be strengthened if controlling for potential confounders (e.g. poverty, literacy rate) and the application of robust estimation strategies to account for unobserved heterogeneity and reverse causality ( Edoka, 2019). One such approach consists of using an instrumental variable (IV) (i.e. a variable that has no direct impact on the health outcome but indirectly, through its impact on health expenditure). IVs should be selected by researchers to fit the local context. One example in the UK has been the use of the ‘funding rule’: local jurisdictions in the UK receive a share of the total budget based on local characteristics, however, the funding rule is revised periodically and this change creates exogenous changes in local funding and generates data for econometric estimation of elasticities ( Claxton *et al., *2018). 

Elasticities will need to be translated into population estimates of cost per DALY averted or QALY gained. If researchers estimated elasticities using mortality data, then those must be converted into QALYs or DALYs using additional assumptions. This requires data on the age and gender structure of the population as well as the morbidity burden of disease. It is worth noting that if the elasticities are estimated from the subset of the population (e.g. children), they will need to be extrapolated for the whole population.

There are several sources of uncertainty attaching to this approach, as there are to other approaches. Given a shortage of vital population statistics, health outcomes are often drawn from survey data, which come with their own shortcomings. Moreover, data on health expenditure is often incomplete (e.g. missing budget items), poorly collected (e.g. inconsistency in recording practice) and unavailable at a low level of aggregation or across several years. There might be uncertainty stemming from the methods employed (e.g. use of an appropriate instrumental variable).

## Discussion

Setting priorities is more than ever before seen as a pre-requisite for achieving global development goals and UHC ( Wiseman *et al.*, 2016). This prerequisite was recognised at the United Nations High-Level Meeting on UHC in 2019 ( United Nations General Assembly, 2019). There is now a push for using HTA across the world to drive more efficient resource allocation in health. Addressing the vacuum left by the abandonment of the 1–3 times GDP per capita CET has therefore become central to determining what services or interventions will be included or excluded within the UHC agenda. In addition, a CET or clear decision rule can signal maturity in resource allocation practices for the health budget to Treasuries or Ministry of Finance. This may support further investments in the health sector, especially in LMICs where health receives low priority within the broader government budgeting process.

This article lays out five alternatives to GDP-based CETs for decision-makers faced with urgent resource allocation decisions, building from our meeting bringing together a selected group of practitioners and researchers. Estimating a health opportunity cost CET using local data is the long-run solution for LMICs since it will explicitly link CETs to budget constraints and help articulate the trade-offs that inevitably arise in coverage decisions. Stakeholders should understand how a CET can be estimated, what the possible alternatives are, and be able to assess the adequacy of the arrangements in, or proposed for, their country. This will require engagement and communication from the onset.

While an empirically estimated supply-side CET using local data, should be the long term aim for all countries, the other four suggestions provide LMICs with tools to structure what is often a difficult ad hoc conversation on value for money and affordability. This intermediate step would represent a significant improvement on current practice and the first four options require no additional or only modest additional resources. In combination, these suggestions can help form deliberative processes that are as evidence-based as possible and that face explicitly up to the complexity of the choices faced by decision-makers ( Baltussen *et al.*, 2016; Chalkidou *et al.*, 2016).

The dangers of applying thresholds that are set too high have been discussed widely elsewhere ( Bertram *et al.*, 2016; Leech *et al.*, 2018; Marseille *et al.*, 2015). It is likely that our proposed approach would lead to more conservative CETs compared to the 1–3 times GDP per capita rule. For this reason, it is worth considering what the implications and risks of *under-estimating* the CET would be. The first obvious consequence would be that cost-effective interventions would be mistakenly ruled out, causing a loss of health at the population level relative to what would have been possible if resources were fully allocated to cost-effective interventions. Some have also argued that a low CET reduces innovation by discouraging manufacturers from seeking to develop new products. Finally, it may be thought that a more conservative CET is incompatible with other social objectives of the healthcare system that do not align or may even conflict with the goal of health maximisation (e.g. priority to the poor or more broadly equity).

These concerns need to be addressed seriously. Further research should help to make more reliable any estimates of a local CET based on health opportunity costs. Further work on the LMIC estimates of Woods *et al.* (2016) and Ochalek *et al*. (2018) would enable better methods and reduce some of the uncertainty and data challenges. Moreover, it is worth noting that CETs are not only used as a simple inclusion/exclusion rule, but also as a basis for price negotiation with manufacturers. In Thailand, economic evaluation has successfully been used to bring down drug prices: for instance, the price of Tenofovir was cut down more than two third from the original to the negotiated price using the CET ( Teerawattananon *et al.*, 2014).

On innovation, there is growing evidence that the vast majority of new products approved for use are only marginal improvements on existing ones, and new market introductions are often priced well above the existing thresholds, especially in LMICs. For example, a discussion of cancer drugs highlights that new introductions are often ‘prohibitively expensive’ and therefore unaffordable for publicly funded systems in LMICs ( Gyawali & Sullivan, 2017). Recent studies have also pointed to the fact that high CETs created perverse incentives on prices, as manufacturers can use the CET to calculate a maximum ceiling price for their products to be accepted ( Gronde *et al.*, 2017). There is no evidence that high aspirational CETs encouraged innovation or access to novel treatments ( Claxton *et al.*, 2009), so the disincentive, if that is what it is, of lower CETs may be similarly unimportant. More important is for LMIC countries individually or collectively to identify the kinds of innovation they would most like to see and then to engage in a discussion with manufacturers and other stakeholders as to suitable incentives (or removal of disincentives). The inclusion of social objectives other than health maximization, like equity, positive discrimination, managerial capacities at various healthcare delivery levels, is independent of the level at which a threshold is set, and should be discussed alongside cost-effectiveness in any HTA framework ( Cookson, 2016). More realistic CETs might well be intrinsically more equitable than high ones because the inclusion of wasted buys leads to crowding out of resources that would otherwise be spent on cost-effective services, usually benefitting the poor.

This piece has provided a menu of options that can be used as an alternative to GDP based thresholds. The primary audience for this piece has been national decision-makers, but our recommendations have ramifications for the global health community. Development partners (DPs) should consider supporting countries in the challenging estimation of locally relevant CETs, working with research institutions with this expertise. The use of CETs to inform resource decisions by DPs has not been widely researched ( Drake, 2014; ( Morton *et al.*, 2018). Should DPs use a single global threshold (as most countries do) or rely on country-specific thresholds, whether estimated by them or the countries in question? This is a contentious issue. Locally estimated CETs reflect the local health opportunity costs, which will be important for countries in transition with increasing co-financing from domestic resources ( Silverman, 2018). This will raise consistency issues if different payers adopt different CETs, or ones that conflict with those preferred by recipient countries. For example, the Global Fund affords priority to the fight against AIDS, Tuberculosis and Malaria, but recipient countries may apply different threshold values to the same programs. On the other hand, using country thresholds would mean that DPs would apply different decision rules to different recipient countries. There is a universal inverse relation between GDP per capita and opportunity cost-based CETs, so using national thresholds may signal a mean that an intervention covered in a middle income country may not be covered in low income one, which may be politically challenging. Conversely, it may also signal to DPs that spending in low income countries is more impactful, as a DALY averted or QALY gain can be realised at lower cost. The matter plainly needs further thought and investigation.

## Data availability

### Underlying data

No data are associated with this article

## Notes

^i ^There is no published review of the use of CETs by decision-makers.

^ii ^For more information about the meeting and its participants, go to: https://www.idsihealth.org/blog/developing-cost-effectiveness-thresholds-to-support-universal-health-coverage/

^iii ^Data limitations are acknowledged in the two papers and use of strong assumptions.

^iv ^Unweighted average

^v ^In preparing this commentary, we searched the cost-effectiveness analyses conducted in LMICs in the last five years using the TUFTS database. Conclusions from this search will be subject to a different piece focusing on the use of CETs in the past five years (forthcoming).
