# Supplementary material for: What next after GDP-based cost-effectiveness thresholds?
Source: Gates Open Res. 2020 Nov 30;4:176. [Version 1] doi: 10.12688/gatesopenres.13201.1 (PMC7851575; doi:10.12688/gatesopenres.13201.1)
Supplement: Peer review history

# Peer review: Reviewer response for version 1

**DOI:** 10.21956/gatesopenres.14409.r30155

**Published:** 2021-02-01

**Authors:** Tharani Loganathan

**Affiliations:** 1 Centre for Epidemiology and Evidence-based Practice, Department of Social and Preventive Medicine, University of Malaya, Kuala Lumpur, Malaysia

Setting a cost-effectiveness threshold is crucial for making the right decisions on allocating limited national resources. Many nations, especially LMICs depend on the 1-3 GDP thresholds for its ease of use and standardisation. This paper reviews the current arguments and details five options to replace GDP based thresholds. At the end of the day, setting priorities are national decisions. And decision rules must be made at the national level, using national data and capacity, while being transparent enough for policy-makers and non-economists to apply with trust. Thus the tools should be simple, relevant and prioritise health systems goals of health maximation and equity while staying within budget.

 Thus as stated, this article is written for an audience of national decision-makers but considers the global health community.

Does the article adequately reference differing views and opinions?

Yes

Are all factual statements correct, and are statements and arguments made adequately supported by citations?

Yes

Is the Open Letter written in accessible language?

Yes

Where applicable, are recommendations and next steps explained clearly for others to follow?

Yes

Is the rationale for the Open Letter provided in sufficient detail?

Yes

Reviewer Expertise:

Vaccine economics

I confirm that I have read this submission and believe that I have an appropriate level of expertise to confirm that it is of an acceptable scientific standard.

# Peer review: Reviewer response for version 1

**DOI:** 10.21956/gatesopenres.14409.r30128

**Published:** 2021-01-13

**Authors:** Christopher James Sampson

**Affiliations:** 1 The Office of Health Economics, London, UK

This article provides a helpful overview of the implications for policymakers of using cost-effectiveness thresholds without relying on GDP-based estimates.

 I have no major criticisms or essential revisions to suggest. My specific points – presented mostly for the purpose of demonstrating that I have fully reviewed the paper – are as follows.

1. In places, the article conflates decisions about new investments, decisions about coverage, and decisions about the optimal allocation of a budget within or beyond health care. The extent to which cost-effectiveness thresholds (as described) can or should inform such decisions is a matter for debate. Therefore, it would be helpful if the article more clearly articulated the types of decisions to which their discussion relates.
2. I believe that the framing of the Options as ‘alternatives’ is unhelpful. Arguably, Options 1 through 4 are – collectively – an alternative to 5. But the most appropriate recommendation is surely that decision-makers consider pursuing all five options rather than just one ‘alternative’. The five options would be better characterised as a to-do list than a menu – as complements, not substitutes – ‘Steps’ or ‘Strategies’ rather than ‘Options’.
3. The authors do identify its limitations, but the article still presents Option 5 as if it is a panacea. Indeed, ‘Options’ 2 and 3 in particular remain relevant even with the realisation of Option 5.
4. The first footnote claims that “There is no published review of the use of CETs by decision-makers.” I’m not sure this is true. I believe that Cameron et al (2018), which is already cited, and Zhang and Garau (2020) do this, at least to some extent. Perhaps the claim is not precisely articulated.

Does the article adequately reference differing views and opinions?

Yes

Are all factual statements correct, and are statements and arguments made adequately supported by citations?

Yes

Is the Open Letter written in accessible language?

Yes

Where applicable, are recommendations and next steps explained clearly for others to follow?

Yes

Is the rationale for the Open Letter provided in sufficient detail?

Yes

Reviewer Expertise:

health economics, cost-effectiveness analysis, health technology assessment, cost-effectiveness thresholds

I confirm that I have read this submission and believe that I have an appropriate level of expertise to confirm that it is of an acceptable scientific standard.

## References

[1] : On what basis are medical cost-effectiveness thresholds set? Clashing opinions and an absence of data: a systematic review. *Glob Health Action*.2018;11(1) : 10.1080/16549716.2018.1447828 1447828 10.1080/16549716.2018.1447828 29564962PMC5930346

[2] : International Cost-Effectiveness Thresholds and Modifiers for HTA Decision Making. *OHE*.2020; Reference source
